# CD44s Assembles Hyaluronan Coat on Filopodia and Extracellular Vesicles and Induces Tumorigenicity of MKN74 Gastric Carcinoma Cells

**DOI:** 10.3390/cells8030276

**Published:** 2019-03-22

**Authors:** Kai Härkönen, Sanna Oikari, Heikki Kyykallio, Janne Capra, Sini Hakkola, Kirsi Ketola, Uma Thanigai Arasu, George Daaboul, Andrew Malloy, Carla Oliveira, Otto Jokelainen, Reijo Sironen, Jaana M. Hartikainen, Kirsi Rilla

**Affiliations:** 1 Institute of Biomedicine, University of Eastern Finland, 70211 Kuopio, Finland; kai.harkonen@uef.fi (K.H.); sanna.oikari@uef.fi (S.O.); heikkiky@student.uef.fi (H.K.); janne.capra@uef.fi (J.C.); sinihak@uef.fi (S.H.); kirsi.ketola@uef.fi (K.K.); uma.thanigai@uef.fi (U.T.A.); 2 Nanoview Nanosciences, Boston, MA 02135, USA; gdaaboul@nanoviewbio.com (G.D.); amalloy@nanoviewdx.com (A.M.); 3 Instituto de Investigação e Inovação em Saúde, University of Porto, 4099-135 Porto, Portugal; carlaol@ipatimup.pt; 4 Institute of Molecular Pathology and Immunology, University of Porto, 4099-135 Porto, Portugal; 5 Department of Pathology and Oncology, Faculty of Medicine, University of Porto, 4099-319 Porto, Portugal; 6 Institute of Clinical Medicine, Clinical Pathology and Forensic Medicine, University of Eastern Finland, 70211 Kuopio, Finland; otto.jokelainen@kuh.fi (O.J.); reijo.sironen@kuh.fi (R.S.); jaana.hartikainen@uef.fi (J.M.H.); 7 Department of Clinical Pathology, Kuopio University Hospital, 70210 Kuopio, Finland; 8 Cancer Center of Eastern Finland, University of Kuopio, 70211 Kuopio, Finland; 9 Translational Cancer Research Area, University of Eastern Finland, 70211 Kuopio, Finland

**Keywords:** CD44, hyaluronan, extracellular vesicle, gastric cancer

## Abstract

CD44 is a multifunctional adhesion molecule typically upregulated in malignant, inflamed and injured tissues. Due to its ability to bind multiple ligands present in the tumor microenvironment, it promotes multiple cellular functions related to tumorigenesis. Recent data has shown that CD44 and its principal ligand hyaluronan (HA) are carried by extracellular vesicles (EV) derived from stem and tumor cells, but the role of CD44 in EV shedding has not been studied so far. To answer this question, we utilized CD44-negative human gastric carcinoma cell line MKN74 manipulated to stably express CD44 standard form (*CD44s*). The effect of CD44s expression on HA metabolism, EV secretion, morphology and growth of these cells was studied. Interestingly, *HAS2* and *HYAL2* expression levels were significantly upregulated in CD44s-expressing cells. Cell-associated HA levels were significantly increased, while HA levels in the culture medium of CD44s-positive cells was lower compared to CD44s-negative MOCK cells. CD44s expression had no significant effect on the proliferation capacity of cells, but cells showed diminished contact inhibition. Superresolution imaging revealed that CD44s and HA were accumulated on filopodia and EVs secreted from CD44s-positive cells, but no differences in total numbers of secreted EV between CD44s-negative and -positive cells was detected. In 3D cultures, CD44s-expressing cells had an enhanced invasion capacity in BME gel and increased spheroidal growth when cultured in collagen I gel. No significant differences in mitotic activity, tumor size or morphology were detected in CAM assays. However, a significant increase in HA staining coverage was detected in CD44s-positive tumors. Interestingly, CD44s-positive EVs embedded in HA-rich matrix were detected in the stromal areas of tumors. The results indicate that CD44s expression significantly increases the HA binding capacity of gastric cancer cells, while the secreted HA is downregulated. CD44s is also carried by EVs secreted by CD44s-expressing cells. These findings highlight the potential usefulness of CD44s and its ligands as multipurpose EV biomarkers, because they are upregulated in inflammatory, injured, and cancer cells and accumulate on the surface of EVs secreted in these situations.

## 1. Introduction

CD44 is transmembrane glycoprotein, also known as homing receptor, that mediates adhesive cell–cell and cell–matrix interactions [1]. The most abundant extracellular matrix (ECM) glycosaminoglycan, hyaluronan (HA) is the main ligand of CD44 [2], but other ECM components including collagen, fibronectin, osteopontin and laminin can also bind CD44 [3].

The extracellular domain of CD44 contains a HA binding site and the cytoplasmic domain is linked to actin cytoskeleton via several proteins, such as ERM (ezrin, radixin and moesin) [4,5]. HA-CD44 interactions induce malignant behavior of tumor cells by activating signaling via several pathways such as via ErbB2 [6], associating with stemness and malignant transformation of cells [3]. It also associates with epithelial-to-mesenchymal transition (EMT) and enhanced migratory and invasive capacity of cancer cells [7].

Upregulation of CD44 is typical for cancer cells and cancer stem cells and is often associated with an unfavorable outcome [8]. CD44s and especially its variant forms act as independent factors for poor prognosis in many cancers originating from epithelial cells [9]. However, the functions of CD44 are controversial since it seems to act both as a suppressor and a promoter of tumor progression, depending on the tumor cell type and the experimental setup [10].

By its high binding affinity to HA, CD44 is involved in the assembly of pericellular HA-rich glycocalyx, which is a typical feature of cells with high HA synthesis activity. HA coat architecture is variable, depending on its assembly by HA chains attached to different HA synthase (HAS) isoenzymes or to CD44 [11,12]. Pericellular HA coat is extremely sensitive for standard formaldehyde fixation methods [13] and is aggregated with methanol [14] or ethanol [15]. This creates challenges for the study design and investigations of HA-CD44 interactions, especially in in vivo experiments.

Because of elevated expression of CD44 in cancer cells and high affinity between CD44 and HA, a variety of different HA-decorated nanocarriers are utilized for targeted cancer therapy [16]. Interestingly, extracellular vesicles (EV), as natural nanocarriers derived from all cell types, are a novel potential source of applications for therapy and diagnostics. EV are membranous cellular fragments that act as multifunctional messengers of the human body in health and diseases such as cancer [17]. The surface composition of EVs is an important feature to regulate EV homing via adhesion and receptor-ligand interactions with target cells. One of the most potential receptors involved is CD44.

In many recent reports, CD44 has been detected in EVs secreted by breast cancer cells [18], stem cells [19,20] and normal cells upon EMT [21]. We have recently reported that HA synthesis induces EV shedding and is accumulated on the EVs of variable size and morphology [19,22,23], and the HA content of EV reflect the content of original cells [19,21]. However, whether *CD44* expression could induce EV secretion activity of cells has not been studied.

The aim of this work was to investigate the effect of *CD44s* expression on the secretion of EVs, HA metabolism, and tumor cell morphology and growth in vitro and in vivo. To answer these questions, CD44-negative human gastric cancer cell line MKN74 was manipulated to stably express CD44 standard form, CD44s, in a bicistronic vector, pIRES-EGFP2. CD44 expression induced a dense HA coat around MKN74 cell filopodia and CD44 was secreted on the surfaces of EVs derived from CD44-positive cells. However, *CD44* expression did not have any effect on the total numbers of EV secreted by gastric cancer cells. Interestingly, *HAS2* and *HYAL2* expression levels were upregulated in CD44s-expressing cells, and total HA levels secreted into the culture media decreased. *CD44s* expression did not affect the mitotic activity of MKN74 gastric cancer cells. However, it induced bigger tumor cell colonies and higher invasion rates, depending on the matrix composition.

Our results suggest that CD44 has a significant effect on the HA content on the surface and between tumor cells, regulating the composition of tumor microenvironment. In addition to its multiple cellular functions, CD44 is both a potential marker but also a functional molecule on the surface of EV, affecting their physical properties, homing, binding and signaling to targets.

## 2. Materials and Methods

### 2.1. Creation of MKN74 Cell Lines and Cell Culture

The CD44s and MOCK versions of the human stomach adenocarcinoma epithelial cell line MKN74 were created and kindly provided by the team of C. Oliveira, a co-author from the Institute of Molecular Pathology and Immunology of University of Porto (IPATIMUP, Portugal). In brief, the sequence coding for *CD44s* (isoform *CD44*-03-ENST00000263398; Ensembl release 68, July 2012) was purchased from OriGene, and further excised and cloned into a pIRES-EGFP2. The correct sequence of this transcript was confirmed by Sanger sequencing. The pIRES-EGFP2 empty vector was used as the MOCK control. Transfection of the vector was performed with Lipofectamine (Life Technologies, Carlsbad, CA, USA). Upon transfection CD44s-expressing cells were cultured in selection medium containing 1 mg/mL of geneticin and subjected to three consecutive rounds of bead sorting. Transfection and selection efficiency was assessed by immunocytochemistry and flow cytometry. MKN74 cells were cultured in RPMI 1640 medium (Lonza, Walkersville, MD, USA) supplemented with 10% FBS (HyClone, Logan, UT, USA), 100 μg/mL streptomycin sulphate (EuroClone, Milan, Italy) and 100 U/mL penicillin (EuroClone, Pero, Italy). For experiments with the EV isolation, FBS was purified by centrifugation at 100,000× *g* for 16 h and sterile-filtered with 0.22 µm syringe filters (Guangzhou Jet Bio-Filtration Co., Ltd., Guangdong, China).

### 2.2. Quantitative Real-Time RT-PCR (qRT-PCR)

Total RNA from the cells were isolated using Tri Reagent (Molecular Research Center Inc., Cincinnati, OH, USA). The cDNAs were synthesized using the Verso cDNA kit (Thermo Scientific, San Jose, CA, USA). The quantitative real-time PCR was performed with Fast Start Universal SYBR Green mix (Roche Applied Science, Indianapolis, IN, USA) using the Stratagene Mx3000P real-time PCR system (Agilent Technologies, Santa Clara, CA, USA). Relative mRNA expression levels were compared by using the 2^−ΔΔC(T)^ method, with Ribosomal protein, Large, P0 (*RPLP0*) as reference gene. The primer sequences are given in Table 1.

### 2.3. Western Blotting

Cells were lysed using RIPA lysis buffer (150 mM NaCl, 50 mM Tris, 1% Nonidet P-40, 0.5% sodium deoxycholate, and 0.1% SDS) containing 10 mM NaF, and 0.5% protease inhibitor cocktail (Sigma–Aldrich, St. Louis, MO, USA). Protein fractions were subjected to 10% SDS-PAGE and transferred to nitrocellulose membranes (Whatman, Sigma–Aldrich, St. Louis, MO, USA), blocked with 3% or 5% bovine serum albumin (BSA) in 1× TBST (Tris-buffered saline containing 0.1% Tween 20) for 1 h at room temperature, followed by overnight incubation with the rabbit polyclonal anti-CD44 antibody (Chemicon, Temecula, CA, USA), diluted in 1% BSA-TBST. After 3 washes with TBST the membranes were incubated for 1 h at room temperature with the infrared secondary antibody, anti-rabbit Dylight 680 (1:5000, Abcam, Cambridge, UK), diluted in 1% BSA-TBST and washed with TBST. The protein bands in the blots were visualized on a LI-COR Odyssey infrared imaging system (LI-COR Biosciences, Lincoln, NE, USA).

### 2.4. Nanoparticle Tracking Analysis

The size distribution and number of the EVs in MKN74 culture media were analyzed with the Nanoparticle Tracking Analyzer (Malvern Instruments Ltd., Malvern, UK) with a NS300 view unit. The conditioned culture media were filtered with 5 µm syringe filter (Sartorius, Goettingen, Germany) to remove cell debris. Filtered media were centrifuged 10,000× *g* for 90 min at 4 °C and the supernatants were centrifuged at 110,000× *g* for 90 min at 4 °C. To collect the whole EV population, pellets from both centrifugation steps were suspended into PBS and combined. The following settings were used for data acquisition: camera level 13, acquisition time 30 s and detection threshold 3. Data analysis was performed with the NTA v3.1 software (NanoSight, Amesbury, UK). Data for each sample was obtained from four replicates.

### 2.5. Nanoview Analysis

Culture media collected from MKN74 cells were utilized for analysis either directly without purification steps or with serial centrifugation as follows: the conditioned culture media were filtered with 5 µm syringe filter (Sartorius, Goettingen, Germany) to remove cell debris. Filtered media were centrifuged at 10,000× *g* for 90 min at 4 °C and the supernatants were centrifuged at 110,000× *g* for 90 min at 4 °C. Pellets from both centrifugation steps were suspended into PBS, combined and diluted 1:20 in PBS before the analysis. EVs were detected using the ExoView chip (ExoView, Boston, MA, USA) arrayed with antibodies against the following proteins: CD81, CD63, CD9, and CD44. Mouse IgG1 was used as negative control. The sample was diluted in PBS with 0.05% tween-20 to get EV concentration within dynamic range of the ExoView assay. Cell culture media and resuspended EV pellet were diluted 3 and 20 times in PBST, respectively. After dilution 35 µL of sample were then incubated on the ExoView Chip placed in a sealed 24 well plate for 16 h at room temperature. The ExoView chips were then washed on an orbital shaker once in PBST for 3 min followed by 3 times in PBS for 3 min. After the PBS washes the chips were rinsed in filtered DI water and dried. The chips were then imaged with the ExoView R100 reader (ExoView, Boston, MA, USA) using the ExoScan v0.998 (ExoView) acquisition software. The data was analyzed using ExoViewer v0.998 with sizing thresholds set to 50 to 200 nm diameter.

### 2.6. Proliferation Rate and Confluency

MKN74 cells were seeded on the 96-well plate (2500 cells/well) and the cells where monitored every 2 h in total of 30 h using Incucyte^®^ S3 Live-Cell Imaging System (Essen BioSciences Ltd., Hertfordshire, UK). The nuclei were stained with IncuCyte^®^ NucLight^®^Rapid Red Reagent (Essen BioSciences, Hertfordshire, UK) and Incucyte S3 2018B software (Essen BioSciences, Hertfordshire, UK) was used to count the numbers of cells and the level of confluency.

### 2.7. HA Assay

Subconfluent cell cultures on 24-well plates were used to measure the HA secretion of MKN74 cells into the culture medium. After treatment or transient transfections, a fresh medium with 5% FBS was changed to the cells, followed by incubation for 24 h before counting the cells and harvesting the media for the sandwich type HA assay as described previously [24].

### 2.8. Immunostaining of CD44 and Staining of HA in Live and Fixed Cells

The cells were cultured on chambered coverglasses (Ibidi GmbH, Martinsried, Germany). For fluorescent stainings, the cells were fixed with 4% paraformaldehyde in phosphate-buffered saline (PBS), pH 7.4 for 20 min. The cells were washed with PBS, permeabilized for 20 min at room temperature with 0.1% Triton-X-100 in 1% BSA and incubated overnight at 4 °C with anti-CD44 monoclonal antibody (1:100, MRQ-13, Cell Marque, Rocklin, CA, USA). After washing, the cells were incubated overnight with fluorescently labelled secondary antibody (l:500; Vector Laboratories Inc., Burlingame, CA, USA). For HA staining, monolayer cultures were incubated overnight at 4 °C with 3 µg/mL of biotinylated HA-binding complex (bHABC). After washing, the sections were incubated for 1 h with Texas red-labelled streptavidin (1:500, Vector, Burlingame, CA, USA). Actin filaments were stained with Phalloidin-iFluor 594 Reagent (Abcam, Cambridge, UK). Nuclei were labeled with 4′,6-diamidino-2-phenylindole (DAPI, 1 μg/mL, Sigma-Aldrich, St. Louis, MO, USA).

### 2.9. Vital Stainings

For staining of pericellular HA coat of live cells, a fluorescently labeled (Alexa Fluor 594) HA binding complex (fHABC) was used as described previously [12]. Live cell cultures grown on chambered coverglasses (Ibidi GmbH, Martinsried, Germany) were incubated for 2 h at 37 °C with 2 μg/mL of fluorescent HABC in culture medium. CellMask™ Deep Red plasma membrane stain (1.25 µg/mL, Molecular Probes, Eugene, OR, USA) was added to the cultures immediately before imaging to label the plasma membranes.

### 2.10. Confocal Imaging and Image Analysis

The fluorescent images were obtained with a Zeiss Axio Observer inverted microscope (40 × NA 1.3 oil or 63 × NA 1.4 oil–objectives) equipped with LSM800 confocal module (Carl Zeiss Microimaging GmbH, Jena, Germany). For live cell imaging, Zeiss XL-LSM S1 incubator with temperature and CO_2_ control was utilized. ZEN v2.3 SP1 (black) software (Carl Zeiss Microimaging GmbH) and ImageJ software (National Institute of Health, Bethesda, MD, USA) were utilized for image processing, 3D rendering and image analysis such as measurements of staining intensity, invasion, spheroidal growth and EV secretion in 3D cultures.

### 2.11. Immuno-Electron Microscopy of EVs

The EV preparations were layered onto carbon-coated glow-discharged copper grids. Grids were fixed in 2% paraformaldehyde for 10 min and contrasted using 2% neutral uranyl acetate for 10–15 min and embedded in 1.8% methyl cellulose (25 Cp)/0.4% uranyl acetate. EV were stained with Monoclonal (mouse) anti-CD44 (1:100, MRQ-13, Cell Marque, Rocklin, CA, USA) and Gold-labelled Antimouse IgG (15 nm gold, Aurion, Wageningen, The Netherlands). Imaging was performed with JEOL JEM 2100F transmission electron microscope (Jeol Ltd., Tokyo, Japan) operated at 200 kV.

### 2.12. Scanning Electron Microcopy

The cells grown on coverslips were fixed with 2% glutaraldehyde and routinely dehydrated in ascending series of ethanol and hexamethyldisilazane and coated with a thin layer of gold. After processing, cells were imaged with a Zeiss Sigma HD|VP (Carl Zeiss Microscopy GmbH, Oberkochen, Germany) scanning electron microscope operated at 3 kV.

### 2.13. Invasion and Spheroidal Assays

To measure the invasion potential, Cultrex Basement Membrane Extract (Trevigen, Gaithersburg, MD, USA) or type I collagen (Trevigen) gel was added on top of confluent MKN74 cultures on 8-well Ibidi chamber slides. For investigation of spheroidal-like growth, cells were mixed with BME or Collagen I gel. The gel was allowed to polymerize at 37 °C for 1 h and the cells were grown for 3 days. The cultures were fixed with 4% paraformaldehyde for 20 min at room temperature. Cultures were imaged with Zeiss LSM800 confocal microscope by utilizing cytoplasmic GFP signal as a marker.

### 2.14. Chick Chorioallantoic Membrane (CAM) Assays

Fertilized white Leghorn chicken eggs were incubated at 37 °C under constant humidity, starting at embryo development day 0 (EDD0). Separation of the CAM was induced on EDD4 by piercing the egg shell. On EDD8 transfected MKN74 CD44-positive cells, and MOCK MKN74 cells were collected, suspended in PBS, and Corning^®^ Matrigel^®^ Matrix GFR Phenol Red Free (Thermo Fisher Scientific Inc., Göteborg, Sweden) (1:1), and implanted on the CAM (10^6^ cells per egg). On EDD13, the tumors were photographed in ovo and excised. Tumor area was measured on photographs from 20 eggs per cell line.

Tumors were fixed in 3% paraformaldehyde, embedded in paraffin and cut in 5 µm sections for assessing the tumor histology by hematoxylin-eosin (HE) staining. The sections were deparaffinized and rehydrated with routine protocols (xylene for 2 × 5 min, absolute EtOH for 2 × 2 min, 94% EtOH for 2 × 2 min), and washed with dH2O for 20 s. The sections were incubated with Delafield’s hematoxylin (Hematoxylin monohydrate (Merck, Darmstadt, Germany)); Supelco Aluminium potassium sulphate dodecahydrate (Merck); AnalaR NORMAPUR ACS Glycerine (VWR International, Leuven, Belgium) for 9 min, and washed with running tap water for 5 min. Excess staining was removed with 1% HCl in 70% EtOH for 4–5 s and washed with running tap water for 10 min. Then the sections were incubated with 1% eosin (Eosin Y GURR, VWR BDH Chemicals) for 30 sec and dehydrated, cleared (94% and absolute EtOH for 2 × 2 min each, xylene for 2 × 5 min), and mounted with DePeX (BDH, Poole, UK).

### 2.15. Staining and Analysis of CAM Tumor Sections

The deparaffinized 4-µm sections were subjected to antigen retrieval by incubation in 10 mM citrate buffer, pH 6.0 for 15 min in a pressure cooker at 120 °C. To block endogenous peroxidase, the sections were treated for 5 min with 1% H_2_O_2_. After washing with 0.1 M Na-phosphate buffer, pH 7.4 (PB), the sections were incubated in 1% bovine serum albumin (BSA) in PB for 30 min to block nonspecific binding. For HA staining, sections were incubated overnight with biotinylated complex of HA-binding region of bovine articular cartilage aggrecan G1 domain and link protein (bHABC) [11] diluted in 1% BSA. For staining of mitotic cells, sections were incubated overnight with primary antibody against proliferation marker protein Ki-67 (Dako, Glostrup, Denmark) and after washing, for 1 h with biotinylated antimouse secondary antibody (1:1000, Vector Laboratories). Stainings were visualized with the avidin–biotin peroxidase method (Vectastain Kit, Vector Laboratories) followed by incubation for 5 min in 0.05% diaminobenzidine (Sigma) and 0.03% hydrogen peroxide in PB, yielding a brown reaction product. The nuclei were stained with Mayer’s hematoxylin. Stained sections were imaged with Zeiss Axio Imager M2 light microscope (Carl Zeiss Microimaging GmbH, Zeiss, Jena, Germany). Stained sections were scanned by Nanozoomer XR digital slide scanner (Hamamatsu Photonics K.K., Hamamatsu City, Japan) at 20× and evaluated by the automated Oncotopix image analysis software v2018.2 (VisioPharm, Hoersholm, Denmark) provided by the Biobank of Eastern Finland.

### 2.16. Statistical Methods

Statistical analyses were carried out using the GraphPad Prism v5.00 for Windows, (GraphPad Software, San Diego, CA, USA). Differences were considered significant when *p* < 0.05.

## 3. Results

### 3.1. Stable CD44s Transfected MKN74 Cells Express High Levels of CD44s

**Figure 1:**
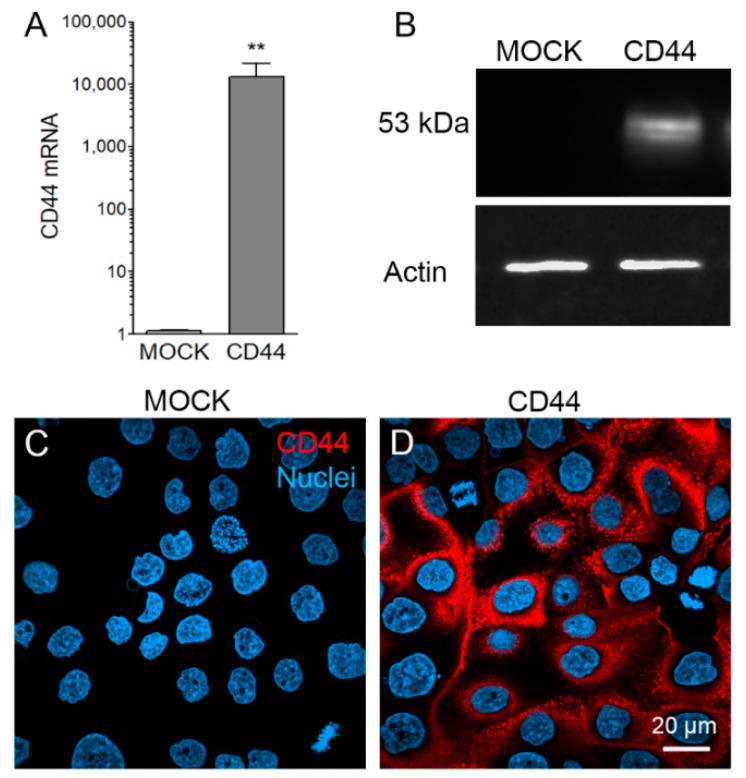
Characterization of *CD44* expression levels in stably transfected MKN74 cells. *CD44* expression levels of MOCK and CD44-positive cells analyzed by QPCR (**A**). Western blot analysis showing the abundance of CD44 proteins in CD44-positive MKN74 cell lines in while MOCK cells are negative (**B**). Immunofluorescence staining of both cell lines with monoclonal CD44 antibody shows the abundant plasma membrane staining of CD44-expressing MKN74 cells (**D**), while MOCK cells are negative for CD44 (**C**). The data represent means ± SE of 6 independent experiments in (**A**), ** *p* < 0.01, Mann–Whitney test.

*CD44* expression levels of two MKN74 cell lines were confirmed with QPCR, Western Blotting and immunofluorescence stainings. QPCR showed extremely low expression levels of CD44 in MOCK cells, while CD44-expressing cells had at least 1000 times higher expression as compared to MOCK cells (Figure 1A). Western blot with CD44 antibody was negative for MOCK cells, while CD44-expressing cells showed a clear level of positivity (Figure 1B). Immunofluorescence staining of monolayer cultures showed no positivity in MOCK cells (Figure 1C), while an intense staining, localized on the plasma membranes was detected in CD44-expressing cells (Figure 1D).

### 3.2. CD44s Regulates HA Metabolism of MKN74 Cell Lines

**Figure 2:**
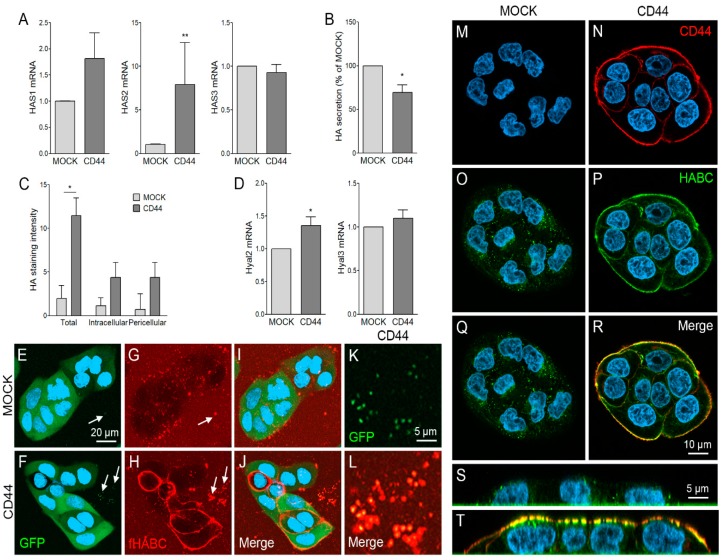
HA metabolism of MOCK and CD44-expressing MKN74 cells. Expression levels of HA synthases (*HAS*1-3) in MKN74 cell lines (**A**). Levels of HA secretion into culture medium (**B**) and the amount of total cell associated, intracellular and surface-associated HA measured as a mean fluorescence intensity of bHABC staining (**C**). Expression levels of hyaluronidases (*HYAL*2 and 3) are shown in (**D**). Maximum projection of confocal optical z-series of live, fHABC-stained MOCK (**E**,**G**,**I**) and CD44-positive (**F**,**H**,**J**) cells. Higher magnification of EVs secreted by CD44-expressing cells are shown in (**K**,**L**). Confocal optical sections of fixed, CD44 (red) and bHABC (AF633 signal pseudocolored as green) stained MOCK (**M**,**O**,**Q**) and CD44-positive (**N**,**P**,**R**) MKN74 cells. Orthogonal sections of MOCK and CD44-positive cells are shown in (**S**,**T**), respectively. The data in (**A**,**B**,**D**) represent means ± S.E. of 6 independent experiments. The means ± S.D. of 3 independent experiments are shown in (**C**). * *p* < 0.05, ** *p* < 0.01; Mann–Whitney test or one-sample *t*-test (**B**).

The levels of three *HAS* isoforms were measured by QPCR. Interestingly, *CD44* expression induced a significant raise in *HAS2* mRNA expression levels (Figure 2A). A moderate, but not statistically significant, increase was also detected in *HAS1*, while *HAS3* levels were unchanged (Figure 2A). HA assay from culture media of MKN74 cells showed a significant decrease in the HA content of the culture media of CD44-expressing cells. Both cell lines secreted moderate amounts of HA, average amount being 2.5 ng/10,000 cells, and 1.9 ng/10,000 cells in MOCK and CD44-positive cells, respectively (Figure 2B). To compare the HA binding capacity of the cells, we analyzed the cell-associated HA levels by measuring the intensity of HABC-reactive staining in fixed monolayer cultures. The total content of cell-associated HA was significantly higher in CD44-expressing cells as compared to MOCK cells (Figure 2C). The intracellular HA was detected by treating the monolayers with hyaluronidase before permeabilization, and pericellular HA was detected by staining non-permeabilized cells. Both intracellular and surface HA levels were higher in CD44-expressing cells as compared to MOCK cells (Figure 2C). The expression levels of *HYAL2* was significantly increased, while no difference was detected in *HYAL3* expression (Figure 2D).

Next, live cells were imaged with confocal microscope to visualize the pericellular HA coats. Signal of GFP was detected to confirm the expression of bicistronic vector in MOCK (Figure 2E) and CD44 (Figure 2F) cells. The HA labeling of live cell cultures showed scattered, spot-like signal on the surfaces of MOCK cells (Figure 2G), while CD44-positive cells had a uniform, highly compact and intense HA layer on their surfaces (Figure 2H). Interestingly, HA-coated EVs attached to the substratum were occasionally found around the cells (arrows in Figure 2E–H). Higher magnification shows the GFP-positive, HA-coated EVs secreted by CD44-positive cells (Figure 2K,L).

We also performed double-stainings for CD44 (Figure 2M,N) and HA (Figure 2O,P) for fixed cells. These stainings showed a colocalization of HA and CD44 in CD44-positive cells (Figure 2R), while only occasional spot-like HABC signal was detected in MOCK cells (Figure 2Q). Orthogonal sectioning of stacks of images showed that the scarce HABC signal of MOCK cells was mostly intracellular (Figure 2S), while most of the HABC signal of CD44-positive cells was located on the plasma membrane, colocalizing with CD44 signal (Figure 2T).

### 3.3. CD44s Accumulates on Filopodia and Binds HA on Filopodial Membranes

**Figure 3:**
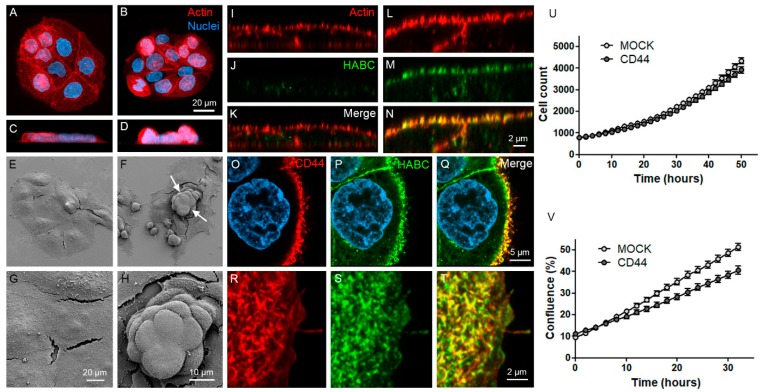
Morphology of MKN74 cells as analyzed by confocal and scanning electron microscopy. Maximum intensity projections created from stacks of confocal optical sections (**A**,**B**) and side views (**C**,**D**) of live MKN74 MOCK (**A**,**C**) and CD44-expressing (**B**,**D**) cells stained with Alexa Fluor 594-labeled phalloidin (red) to label the actin cytoskeleton. Scanning electron microscopy of MOCK (**E**,**G**) and CD44 (**F**,**H**) cultures with low (**E**,**F**) and high (**G**,**H**) magnification. Superresolution orthogonal sections of MOCK (**I**–**K**) and CD44 (**L**–**N**) cells double-stained with actin (**I**,**L**) and HA (**J**,**M**). Merged images are shown in (**K**,**N**). Superresolution sections of CD44-positive cells double-stained with CD44 (**O**) and bHABC (**P**) and maximum intensity projections from the surface of the same cell with CD44 (**R**) and bHABC (**S**). Merged images from (**O**,**P**) and (**R**,**S**) are shown in (**Q**,**T**), correspondingly. Incucyte live cell analysis of proliferation rate (**U**) and confluency (**V**) of the cells. Data are representative of 3 independent experiments. The means ± S.E of 4 replicates are shown in (**U**,**V**).

The morphology of MKN74 MOCK and CD44-expressing cells was studied by confocal and scanning electron microscopy. Staining of actin cytoskeleton by phalloidin staining indicated that CD44-positive cells had a more rounded morphology (Figure 3B,D) as compared to MOCK cells (Figure 3A,C). The different morphology is particularly clearly seen in the side views of MOCK (Figure 3C) and CD44-expressing (Figure 3D) cells. This was confirmed by SEM imaging, which also showed the same effect and revealed that CD44-positive cells tended to grow on the top of each other, forming tightly packed cell clusters (arrows in Figure 3F). A more detailed view of MOCK (Figure 3G) and CD44 (Figure 3H) surface morphology shows high numbers of tiny filopodia on the surface of both cell lines. Double-staining of actin and HA imaged by superresolution (Airyscan) indicated a strong localization of HA with the plasma membrane filopodia of CD44-expressing cells (Figure 3N), while only sparse spot-like HA staining was detected in MOCK cells (Figure 3J). Superresolution imaging of CD44 and HABC double-staining of CD44-positive cells showed that CD44 was also localized and accumulated in filopodia colocalizing with HA, as demonstrated in optical sections (Figure 3O–Q), and in maximum intensity projection from a small area on the cell surface (Figure 3R–T). Incucyte live cell analysis showed no difference in the proliferation rate of the two cell lines (cell counts, Figure 3U). However, MOCK cells reached a higher level of confluency (Figure 3V), indicating a more rounded morphology of CD44-positive cells.

### 3.4. CD44s Is Accumulated on EVs but Does Not Affect EV Secretion Levels

**Figure 4:**
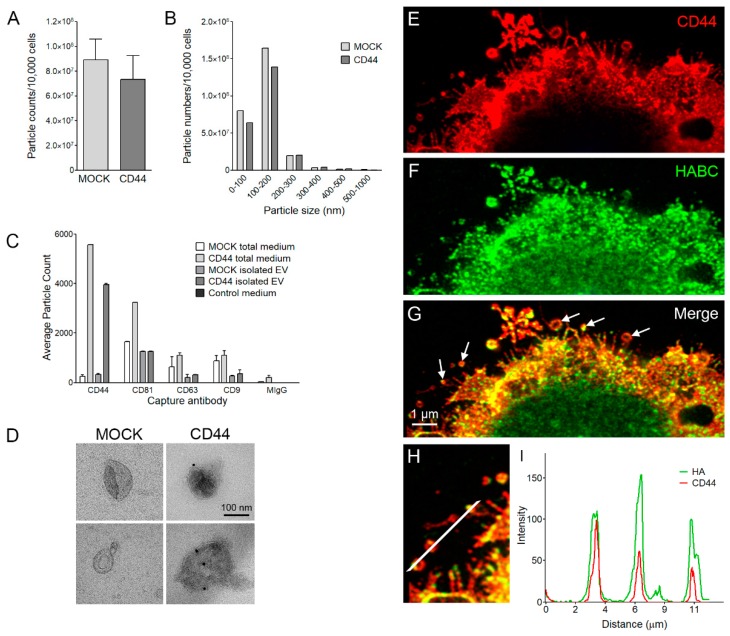
MKN74 cell EV secretion levels and CD44 carried by EVs. Particle counts normalized to cell numbers (**A**) and size distribution of particles (**B**) as measured by NTA. The means ± S.D. of three independent experiments are shown in (**A**). ExoView analysis of CD44, CD81, CD63 and CD9 -positive EV directly from the conditioned culture media of MKN74 cells and from the isolated EV preparations (**C**). Immuno-electron microscopy of isolated EV preparations labeled with CD44 antibody with two examples of EV derived from both cell lines (**D**). Superresolution optical sections from plasma membrane area of a CD44-expressing cell secreting CD44 (**E**) and HA (**F**) -positive EV. Merged image is shown in (**G**). Intensity profile through three EV shown in panel (**H**) is represented in (**I**).

Next, the secretion and size distribution of EVs secreted by both cell lines was analyzed by NTA analysis, which did not detect any significant differences in the total particle numbers (Figure 4A). The size distribution of EVs was similar in both cell lines (Figure 4B), the average diameter being 135 ± 9 nm in MOCK and 143 ± 10 nm in CD44-positive cells. Next, the vesicle populations were characterized with ExoView affinity microarray platform analysis. The high CD44 positivity of EV secreted from CD44-expressing cells was clearly seen with CD44 antibody, both from unpurified culture medium as well as from isolated preparation (Figure 4C). EVs from both cell lines were captured by general EV markers CD81, CD62 and CD9 (Figure 4C). Immuno-EM of isolated EV preparations confirmed the CD44-positivity of EV derived from CD44-positive cells (Figure 4D). Next, the CD44-expressing cell cultures were imaged by super-resolution microscopy to detect EVs in situ on or near the plasma membrane of secreting cell. Superresolution microscopy revealed that CD44 (Figure 4E) and HA (Figure 4F) were localized on EVs of variable size, budding from the plasma membranes and filopodial tips of cells (arrows in Figure 4G). Intensity profiling (Figure 4H) showed a colocalization of CD44 and HA signals in EVs (Figure 4I).

### 3.5. CD44s Induces Growth and Invasion but Not EV Secretion by MKN74 Cells in 3D Collagen Gels

**Figure 5:**
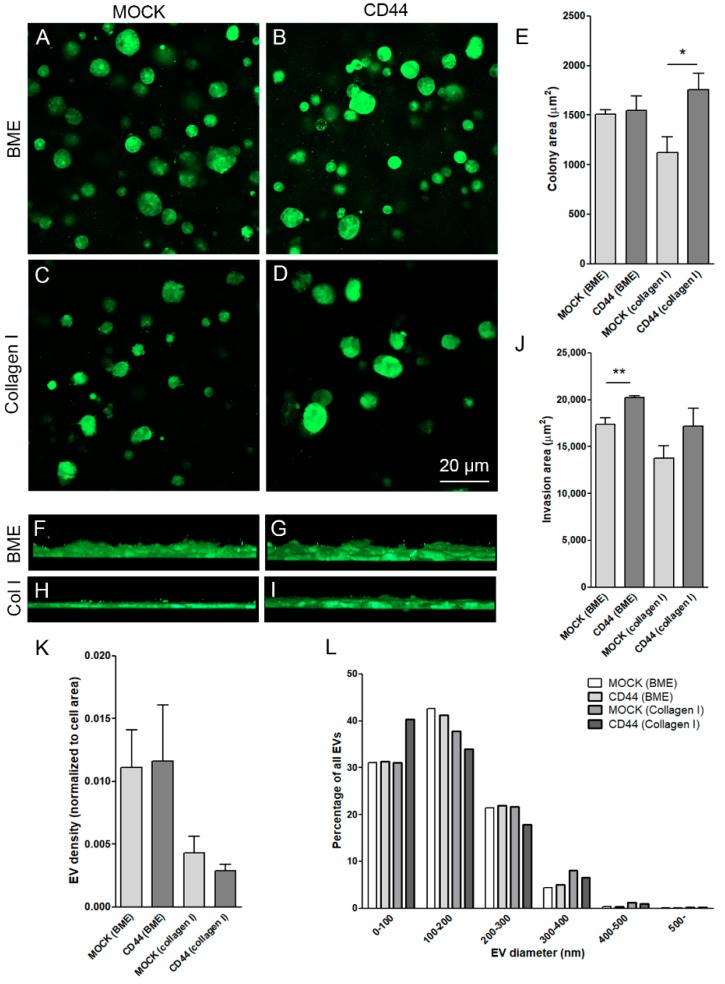
Colony formation and invasion rate of MNK74 cells grown in collagen I and BME gels. Confocal optical sections of MOCK (**A**,**C**) and CD44-positive (**B**,**D**) cells grown in BME (**A**,**B**) and collagen I (**C**,**D**) gels. The areas of colonies in all groups were measured from optical sections (**E**). Side views of invasion areas of MOCK (**F**,**H**) and CD44-positive (**G**,**I**) cells grown in BME (**F**,**G**) and collagen I (**H**,**I**) gels. Number of secreted EV into the matrix of 3D cultures, normalized to cell area (**K**) and EV size distribution are shown in (**L**). GFP was used as a marker to visualize the cells and EVs. The data represent means ±S.E. of 4–5 independent experiments. * *p* < 0.05, ** *p* < 0.01; *t*-test.

We utilized two different matrices, BME gel (rich with collagen IV) and collagen I gel, to study the spheroid-like growth and invasion capacity of MKN74 cells. The cells formed spheroidal colonies of variable size in both matrices (Figure 5A–D). When grown in BME gel, there was no difference in the average sphere areas between MOCK and CD44-positive cells (Figure 5E), while in collagen I, CD44-expressing cells formed bigger spheres (Figure 5E). Next the invasion capacity of MKN74 cells was analyzed by seeding the cells under the collagen gels. The overall invasion capacity of MKN74 cells was relatively low during the 48-h culture period (Figure 5F–J). There was a higher tendency of CD44-positive cells for invasion, but the difference was statistically significant only when cultured under BME gel (Figure 5J). The EV secretion was analyzed from the same cultures by counting the GFP-positive particles embedded in the matrix. Because of different cell numbers in cultures, the EV numbers were normalized to the area covered by cells (Figure 5K). No significant differences in EV shedding between MOCK and CD44-expressing cells was detected. However, EV secretion was higher when the cells were grown in BME gel as compared to collagen I gel (Figure 5K). The size distribution of EVs in 3D cultures was similar to monolayer cultures (see Figure 4B). The average diameter of EVs was 149 ± 0.7 nm in MOCK and 151 ± 0.8 nm in CD44-positive cells in BME gel, and 159 ± 1.2 nm in MOCK and 155 ± 1.3 nm in CD44-positive cells in collagen I gel. No differences in size distribution of EVs was detected (Figure 5L).

### 3.6. CD44s Expression Does Not Affect the Growth and Other Features of CAM Tumors

**Figure 6:**
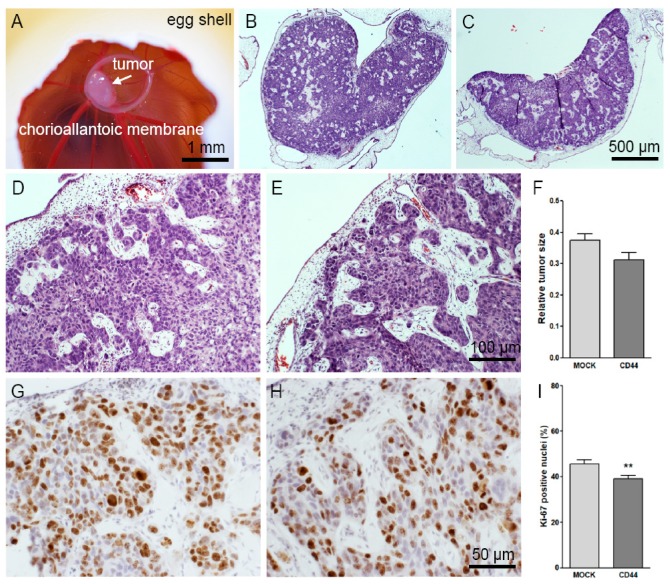
Analysis of growth, morphology and proliferation of MKN74 tumors in CAM assays. A typical example of MKN74-derived CAM tumor (EDD13) is shown in (**A**). Whole sections of HE-stained tumors from MOCK and CD44-positive cells are shown in (**B**,**C**), correspondingly. Higher magnification images from HE-stainings show the overall morphology of MOCK (**D**) and CD44-positive (**E**) tumor tissues. The relative tumor size of both groups is shown in (**F**). Representative images of Ki-67-stainings (brown) of MOCK (**G**) and CD44-positive (**H**) tumor sections. The proportion of Ki-67-positive nuclei in both tumor groups is shown in (**I**); *n* = 20 in both groups. ** *p* < 0.01.

MKN74 cells transplanted onto the CAM membrane of fertilized eggs resulted in rapid tumor formation (Figure 6A–C). The tumors were formed by islands and chords formed by highly atypical epithelial cells. Occasional glandular structures were observed among otherwise diffuse growth. Formation of active connective tissue stroma with spindled fibroblastic cells and a rich neovascular component was also evident (Figure 6B,C). No obvious mucinous component was produced. These features are typical for poorly differentiated invasive adenocarcinoma (Figure 6D,E). Tumor sizes were estimated by measuring and correlating the areas of fresh, in ovo-photographed tumors to the area of plastic ring, which was known to be constant (Figure 6A). No significant differences in the size of MOCK and CD44-positive tumors were detected (Figure 6F). Some infiltrative tumor cell chords were noticed at the invasive front, but no metastases or intravascular invasion into native capillaries of the CAM membranes were detected. The histopathological features of MOCK and CD44 tumors were similar in HE stained slides (Figure 6E,F). The mitotic activity of tumor cells was analyzed by counting the proportion of Ki-67 positive nuclei (Figure 6G,H). The numbers of Ki-67-positive cells were slightly but significantly decreased in CD44-expressing tumors (Figure 6I).

### 3.7. CD44s Increases HA Content of the CAM Tumors

**Figure 7:**
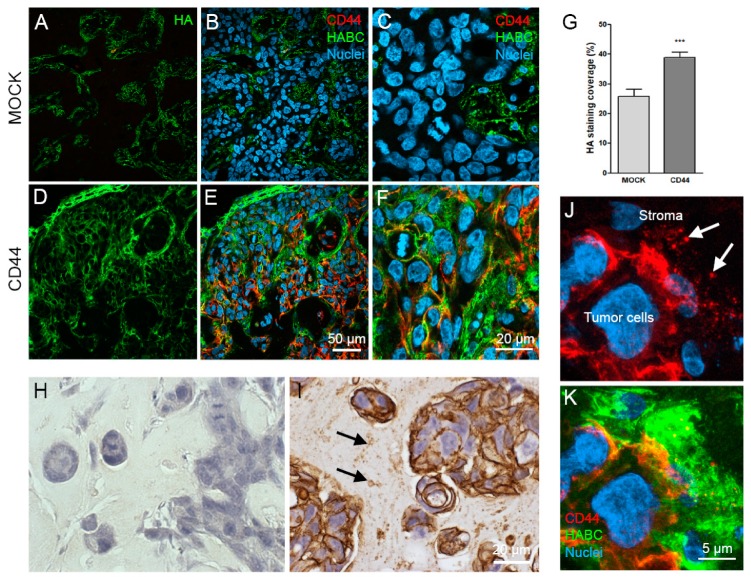
HA and CD44 content of CAM tumors. Confocal optical sections from CAM tumor paraffin sections derived from MOCK (**A**–**C**) and CD44-positive (**D**–**F**) cells, double-stained for HA (**A**,**D**, green) and CD44 (**B**,**C**,**E**,**F**, red, merged with green). The HA staining coverage visualized with DAB was analyzed from paraffin sections (**G**); *n* = 20 in both groups. CD44 (DAB, brown) staining of MOCK (**H**) and CD44-positive (**I**) tumors and superresolution 3D projections from CD44-positive tumors (**J**,**K**), double-stained with bHABC (green) and CD44 antibody (red). Arrows in (**I**) indicate the CD44-positive EVs in stromal area. *** *p* < 0.001.

CAM tumor paraffin sections were double-stained with CD44 antibody and HABC to analyze the amount and localization of CD44 and HA (Figure 7). HA was accumulated mainly in the stromal areas of the tumors formed by MOCK cells (Figure 7A–C), while tumor cells were negative. In CD44-positive tumors, HA staining was intense around and between tumor cells, colocalizing with the plasma membrane CD44 signal (Figure 7D–F). The total HA staining coverage of tumor sections was analyzed, and there was a significant increase in the intensity of HA staining in CD44-positive tumors as compared to MOCK tumors (Figure 7G), which is in line with the results of the cell cultures. Interestingly, CD44 staining of CD44-expressing tumors revealed a high number of EVs of different size in the stromal areas (Figure 7I, arrows), while the tumor cells as well as the stroma of MOCK tumors were completely negative for CD44 immunostaining (Figure 7H). Superresolution 3D confocal imaging of CD44-HA double-stainings confirmed the CD44-positivity of stromal EV and indicated that they were surrounded by high amounts of HA (arrows in Figure 7J,K).

## 4. Discussion

### 4.1. Characterization of MNK74 Cells with CD44 Expression

The adhesion receptor CD44 contributes to various diseases, the most widely studied being various cancers. Despite of extensive research the exact role of CD44s and its numerous variant forms in malignancy is still controversary [10]. The novel research area with growing recent interest is the role of CD44 in EV biology. This study was focused on standard form of CD44 and its effect on morphology, malignant potential, HA metabolism and EV secretion of gastric carcinoma cells.

### 4.2. Effect of CD44 Expression on HA Metabolism

To avoid the possible interference of GFP-tag on the functionality of CD44, the bicistronic expression of GFP and CD44 was utilized to express CD44s in CD44-negative gastric carcinoma cell line. *CD44s* expression in CD44-negative gastric cancer cells with moderate HA secretion levels resulted in significant increase in cell-associated HA. Interestingly, CD44 expression decreased the HA content of culture media of MKN74 cells. This is probably due to increased HA binding to the plasma membranes via CD44 which resulted in lower soluble HA content in culture medium.

Intracellular HA levels were also increased in CD44-expressing cells, suggesting that increased binding of HA on the plasma membranes may have resulted in enhanced endocytosis and subsequent degradation of HA [25,26]. Expression of *HYAL2* was also significantly induced in CD44-expressing cells, which could have accelerated the degradation rate of HA. This may explain why the HA content in the culture medium did not increase despite of significant upregulation of *HAS*2 expression in CD44-expressing cells. Another explanation may be a tight CD44-dependent HA coat on the plasma membrane that may have blocked HAS insertion into the plasma membrane, inhibiting its activity [27]. The upregulated *HAS2* levels upon *CD44* expression suggest that *CD44* and *HAS2* are reciprocally regulated, which is in line with previous studies [28]. It is also possible that enhanced CD44-HA signaling induces a positive feedback [29] to enhance HAS expression. These results indicate the potential of CD44 in modulating the HA metabolism not just via binding of HA but also in the level of synthesis and degradation. In many cases, HAS activity is the primary factor that impacts HA accumulation into tumor tissue [30]. Overall, our results showed that despite of the relatively low HA secretion levels of MKN74 tumor cells, CD44 has a significant effect on the HA content, regulating the pericellular HA assembly on the surface and between tumor cells.

### 4.3. Structure of HA Coat Induced by CD44

Interestingly, the HA coat induced by *CD44* expression was highly resistant for fixation. Usually HA coat is affected by different fixation methods [13] and HA content is often underestimated in fixed samples [15]. A recent study has shown that bonds between HA and CD44 are remarkably strong [31], which may explain the permanence of the coat around CD44-expressing cells.

The HA coat induced by CD44s around MNK74 cells was uniform, highly compact and intense in structure. Typically, the size of the pericellular HA coat correlates with activity of HA synthesis [32]. Furthermore, the structure of the coat varies depending on expression levels of *HAS* isoforms. HAS1 induces a faint HA coat which is dependent on CD44 [33], while the coat induced by HAS2 and HAS3 is tight and intense [34] and scaffolded by long filopodia, especially in *HAS3*-expressing cells [12]. Additionally, HA binding proteins, such as aggrecan are also able to modify and expand the coat structure, having an essential functional role especially in cartilage [35].

We showed here that CD44s was especially accumulated into very short filopodia of MKN74 cells, but actin stainings showed similar filopodia in CD44-negative cells. This indicates that despite its ability to assemble a tight HA layer around filopodial membranes, CD44s expression does not induce filopodial growth. Existence of filopodia in CD44-negative MOCK cells indicates that CD44 is not essential for filopodial formation, which is in line with previous reports [36,37].

### 4.4. CD44-Expressing MKN74 Cells Secrete EVs That Carry CD44 and HA

We have shown that increased HA synthesis activity by HAS3 [22] and HAS2 [38] induces secretion of EV, which is regulated by HAS activity [22] or HAS residence in plasma membrane [38]. However, the results of this work indicated that CD44 expression does not induce the total number of secreted EV of gastric cancer cells. In cells with high HAS activity, HA on the surface of EV can be attached to HAS, independent of CD44 [22]. According to our recent findings in primary cells, HA content of secreted EVs reflects HA synthesis activity of original cell [19,21]. The results of this work show that CD44 was carried on the surfaces of EVs secreted by CD44-expressing cells, but was not detectable on EVs derived from MOCK cells. In some previous reports, CD44 was detected in cancer cell-derived EV [18,39]. It may have a role on binding HA not just on the cellular surfaces, but also on secreted EVs. Also, HA was detected on the surfaces of EV, which shows that CD44 can assemble a HA coat around EV membrane. These findings emphasize the potential role of CD44 as an EV biomarker.

CD44 is known as a ‘homing receptor’, because HA-CD44 interactions mediate the recruitment of activated leucocytes [40], stem cells [41] and tumor cells [42] from the circulation. The strong bonds between HA and CD44 [31] provide resistance to shear during primary adhesion of lymphocytes on vascular endothelial cells [43]. The same mechanism potentially regulates the homing of circulating EV that reprogram the tumor supporting cells at a target site to prepare a favorable environment for cancer cells. There is evidence that CD44 expressed on the EV membranes may promote EV-mediated metastases of ovarian to the mesothelial cells of the abdominal cavity, promoting metastasis [39]. This is highly interesting, because our recent study showed that EMT is associated with CD44 expression and enhanced secretion of EV by mesothelial cells [44]. Detection of CD44-positive EVs would be a promising tool for diagnostics of cancers with changed CD44 expression. Furthermore, blocking of the CD44–HA interactions between cells [8] but also between cells and EVs can offer new treatment options for cancer.

CD44-HA interactions have also been utilized in targeting of liposomes [45] and drug conjugates [46] and enhancing cellular uptake of nanoparticles [47]. The high expression of CD44 in tumors compared with normal cells potentially enhances uptake of HA-positive vesicles via HA-CD44 pathways [16]. In addition to its multiple cellular functions such as ligand binding, induction of malignancy, lymphocyte homing and tumor cell homing, CD44 is a potential functional molecule on the surface of EVs, effecting their physical properties, homing, binding and signaling to targets and biomarker for circulating tumor-derived EVs. In the future it will be necessary to study the impact of CD44 on the mechanisms of homing and binding of EV to the target cells.

### 4.5. Association of CD44 with Tumorigenic Properties

Several studies have reported that CD44-positivity is significantly associated with poor survival in gastric cancer patients [48,49,50], and presence of CD44-positive cancer stem-like cells especially at the invasive tumor front [51] act as an indicator of poor prognosis. The results of this study showed that CD44s expression does not induce proliferation rate of MKN74 cells and had no effect on the resulting tumor size. However, the tumor cell-associated HA levels were significantly increased in CD44s-positive tumors. Changes in CD44 expression are typically one of the latest manifestations in the malignant development of gastric adenocarcinoma [52].

However, the overall role of CD44 in cancer is conflicting. CD44 correlates with HA staining in many cancers, such as squamous cell carcinoma and malignant melanoma [53]. Despite of clear poor prognostic value of CD44 in tumors such as breast [54] and renal cell [55] carcinomas, CD44 expression is a favorable predictor in ovarian cancer [56,57]. Furthermore, reintroduction of CD44 to highly tumorigenic CD44-deficient fibroblasts reduced their tumorigenic properties [58].

CD44-induced invasion and spheroidal growth of MKN74 were dependent on surrounding matrix composition. In real life, the composition of the ECM is more complex [59] and multiple binding partners of CD44 may enhance this complexity. According to these findings we can conclude that the conflicting effects of CD44 on cancer cell properties are, at least partly, due to microenvironmental factors such as composition of the matrix, genetic background of cancer cells [60], ER status [28] and cancer origin [61]. In this work we focused on CD44s expression, but several diverse variants of CD44 exert a wide spectrum of functions [8]. Taken together, this study shows that CD44 has an important role in arresting HA around tumor cells, having a significant effect on tumor cell HA content independent on levels of HA synthesis. Its effects are also potentially mediated through modification of tumor microenvironment via its high affinity to HA and other extracellular ligands. The effect of matrix composition in EV shedding, not dependent on CD44s expression, was an interesting finding and enhances the importance of ECM in EV biology [62].

## 5. Conclusions

The results of this study indicate that CD44s is a strong regulator of gastric carcinoma HA metabolism via effecting the expression levels of HA synthesizing and degrading enzymes. It also enhances HA binding capacity of the cells and modulates the morphology and invasive capacity of cells. CD44s expression does not have impact on the total EV secretion activity of tumor cells, but it is carried and easily detected on EVs. Because of high affinity to HA, CD44 carried by the EVs has potential in regulating EV interactions with the ECM. The present study extends knowledge of the role of CD44 in cancer cell biology. As an easily detectable surface molecule carried on the EV and their donor cells, CD44 has potential as a biomarker in cancer and other conditions associated with enhanced CD44 expression such as inflammation.

## Figures and Tables

**Table 1:** Quantitative real-time RT-PCR primer sequences.

Gene		Primer Sequences	
**Hyaluronan synthase 1**	*HAS1*	Forward 5′	CAAGATTCTTCAGTCTGGAC
		Reverse 5′	TAAGAACGAGGAGAAAGCAG
**Hyaluronan synthase 2**	*HAS2*	Forward 5′	CAGAATCCAAACAGACAGTTC
		Reverse 5′	TAAGGTGTTGTGTGTGACTG
**Hyaluronan synthase 3**	*HAS3*	Forward 5′	CTTAAGGGTTGCTTGCTTGC
		Reverse 5′	GTTCGTGGGAGATGAAGGAA
**CD44**	*CD44*	Forward 5′	CATCTACCCCAGCAACCCTA
		Reverse 5′	CTGTCTGTGCTGTCGGTGAT
**Hyaluronidase 2**	*HYAL2*	Forward 5′	CCTCTGGGGCTTCTACCTCT
		Reverse 5′	CTGAACACGGAAGCTCACAA
**Hyaluronidase 3**	*HYAL3*	Forward 5′	GCTGGCATAGTATGGCTTCC
		Reverse 5′	ACACCAATGGACTGCACAAG
**Ribosomal protein, Large, P0**	*RPLP0*	Forward 5′	AGATGCAGCAGATCCGCAT
		Reverse 5′	GTGGTGATACCTAAAGCCTG

## Abbreviations

BME	Basement membrane extract
CAM	Chick chorioallantoic membrane assay
CD44	Cluster of differentiation 44
ECM	Extracellular matrix
EMT	Epithelial-to-mesenchymal transition
EV	Extracellular vesicle
HA	Hyaluronan
bHABC	Biotinylated hyaluronan binding complex
HAS	Hyaluronan synthase
HYAL	Hyaluronidase
NTA	Nanoparticle tracking analysis
